# Decentralized EEG-based detection of major depressive disorder via transformer architectures and split learning

**DOI:** 10.3389/fncom.2025.1569828

**Published:** 2025-04-16

**Authors:** Muhammad Umair, Jawad Ahmad, Nada Alasbali, Oumaima Saidani, Muhammad Hanif, Aizaz Ahmad Khattak, Muhammad Shahbaz Khan

**Affiliations:** ^1^Faculty of Engineering, Multimedia University, Cyberjaya, Malaysia; ^2^Cybersecurity Center, Prince Mohammad Bin Fahd University, Al-Khobar, Saudi Arabia; ^3^Department of Informatics and Computer Systems, College of Computer Science, King Khalid University, Abha, Saudi Arabia; ^4^Department of Information Systems, College of Computer and Information Sciences, Princess Nourah bint Abdulrahman University, Riyadh, Saudi Arabia; ^5^Department of Informatics, School of Business, Örebro Universitet, Örebro, Sweden; ^6^School of Computing, Engineering and the Built Environment, Edinburgh Napier University, Edinburgh, United Kingdom

**Keywords:** split learning, transformers, autoencoder, EEG, major depressive disorder, smart diagnostic, neurological behavior

## Abstract

**Introduction:**

Major Depressive Disorder (MDD) remains a critical mental health concern, necessitating accurate detection. Traditional approaches to diagnosing MDD often rely on manual Electroencephalography (EEG) analysis to identify potential disorders. However, the inherent complexity of EEG signals along with the human error in interpreting these readings requires the need for more reliable, automated methods of detection.

**Methods:**

This study utilizes EEG signals to classify MDD and healthy individuals through a combination of machine learning, deep learning, and split learning approaches. State of the art machine learning models i.e., Random Forest, Support Vector Machine, and Gradient Boosting are utilized, while deep learning models such as Transformers and Autoencoders are selected for their robust feature-extraction capabilities. Traditional methods for training machine learning and deep learning models raises data privacy concerns and require significant computational resources. To address these issues, the study applies a split learning framework. In this framework, an ensemble learning technique has been utilized that combines the best performing machine and deep learning models.

**Results:**

Results demonstrate a commendable classification performance with certain ensemble methods, and a Transformer-Random Forest combination achieved 99% accuracy. In addition, to address data-sharing constraints, a split learning framework is implemented across three clients, yielding high accuracy (over 95%) while preserving privacy. The best client recorded 96.23% accuracy, underscoring the robustness of combining Transformers with Random Forest under resource-constrained conditions.

**Discussion:**

These findings demonstrate that distributed deep learning pipelines can deliver precise MDD detection from EEG data without compromising data security. Proposed framework keeps data on local nodes and only exchanges intermediate representations. This approach meets institutional privacy requirements while providing robust classification outcomes.

## 1 Introduction

The human body possess remarkable complexity, and the brain plays a pivotal role in cognitive and behavioral functions (Vohryzek et al., 2025). Maintaining a healthy brain is essential for optimal decision-making (Hagan et al., 2025). The human brain contains billions of neuron, which coordinate various neurological activities (Herculano-Houzel, 2009). Nonetheless, a range of disorders impact brain function, including Major Depressive Disorder (MDD) i.e., leading contributor to mental health challenges (Kreivinienė et al., 2025).

Early diagnosis of MDD is important for mentaining mental well-being, but current diagnostic methods rely on subjective clinical evaluations and self-reported symptoms prone to human error and inefficiency (Hagan et al., 2025; Kreivinienė et al., 2025). This underscores the need for a reliable diagnostic tool that assists clinicians in making accurate and timely decisions.

Electroencephalography (EEG) offers a promising approach for examining the neurophysiological underpinnings of mental health conditions (Perrottelli et al., 2021). It measures electrical brain activity with high temporal resolution and is non-invasive, cost-effective, and portable (Perrottelli et al., 2021). Previous studies have revealed changes in EEG patterns, such as power spectral density shifts and alterations in brain wavebands, among individuals with MDD (Liang et al., 2021). Although EEG signals contain valuable diagnostic information, extracting meaningful insights from these high-dimensional and noisy data remains a challenge.

Machine learning (ML) and deep learning (DL) techniques demonstrate potential for analyzing EEG signals (Subhani et al., 2017; Rahul et al., 2024; Umair et al., 2021; Diehl and Cook, 2015). DL models can automatically extract relevant patterns, aiding in differentiating healthy individuals from those affected by MDD (Subhani et al., 2017). However, traditional ML and DL training often occurs in centralized systems, which raises privacy risks and demand costly computational infrastructure (Umair et al., 2024; Rahul et al., 2024). Healthcare institutions also hesitate to share sensitive data, highlighting the need for decentralized methods that safeguard patient privacy (Umair et al., 2023).

Federated Learning (FL) has emerged as a key approach to decentralized training by enabling local model updates on client devices while aggregating models at a central server (McMahan et al., 2017). Although FL preserves data privacy, some clients may face resource constraints that hinder local training (Umair et al., 2023). However, a similar concept as FL i.e., split learning (SL) addresses this challenge by splitting the model architecture between clients and a central server, transferring only intermediate representations instead of raw data (Gupta and Raskar, 2018). This structure reduces the computational burden on resource-limited devices having on device training as well (Jia et al., 2024). In the context of EEG-based MDD diagnosis, SL can integrate distributed data from multiple healthcare providers without centralizing sensitive information, offering a scalable and reliable framework for developing effective diagnostic models.

This study explores the concept of SL in conjunction with various ML and DL models to classify MDD patients using an EEG dataset. Model selection is critical for robust classification, so multiple ML classifiers including Logistic Regression (LR), Random Forest (RF), Support Vector Machine (SVM), Decision Tree (DT), K-Nearest Neighbors (KNN), and Gradient Boosting (GB) are utilized for their proven performance. In addition, advanced DL architectures such as Transformers and Autoencoders are employed to capture the complex, high-dimensional characteristics of EEG data. An ensemble learning principles is then implemented in a SL framework, with three clients chosen for comparative evaluation. Classification reports and confusion matrices serve as the primary metrics to assess the performance of these models. Thus, key contribution of this study is as follows:

1. Split learning framework tailored for EEG-based MDD classification. And within this SL approach ML and DL models are utilized for EEG features extractions and classification.
2. Proposed a ensemble model tailored for MDD disorder classification through comprehensive performance metrics across three clients in SL settings.

This article is organized into five main sections. Section 1 provides the background and context of the study. Section 2 reviews related work and relevant literature. Section 3 details the methods and materials used in the experiments. Section 4 presents the obtained results and offers a comprehensive discussion. Finally, Section 5 concludes the study by summarizing the key findings.

## 2 Related work

Researchers have recently explored a range of ML and DL models for medical applications (Gour et al., 2023; Sultan et al., 2023; Owais et al., 2022) yielding promising results. However, as discussed in Section 1, the majority of these algorithms rely on centralized architectures that raise privacy concerns and limit their practical applicability. This section reviews recent studies that utilize ML and DL approaches for EEG-based analysis, as well as decentralized solutions aimed at safeguarding data privacy and promoting scalability.

Park et al. (2021) employed multiple ML models SVM, RF, and elastic net regression to classify six major psychiatric disorders and healthy controls using EEG features such as power spectrum density (PSD) and functional connectivity (FC). Their elastic net model achieved the highest accuracy across disorders, notably identifying schizophrenia with 93.83% accuracy using alpha PSD, anxiety disorders with 91.03% accuracy via whole-band PSD, and trauma and stress-related disorders with 91.21% accuracy from beta FC features. Rafiei et al. (2022) proposed a DL model based on a customized InceptionTime architecture for MDD detection, achieving 91.67% accuracy with full-channel EEG data and 87.5% after channel reduction. Rivera et al. (2022) conducted a systematic mapping of 46 primary studies that leveraged DL for EEG-based mental disorder diagnoses, revealing CNNs as the most common approach and epilepsy as the most frequently studied disorder. Wang et al. (2024) developed DiffMDD, a diffusion-based DL framework for diagnosing MDD, incorporating Forward Diffusion Noisy Training and Reverse Diffusion Data Augmentation to mitigate noise and data scarcity. Anik et al. (2024) introduced an 11-layer 1D-CNN for MDD classification, focusing on gamma band EEG segments of 15-second epochs, and attained 99.60% accuracy, 100% sensitivity, and 99.21% specificity.

Earl et al. (2024) used an RF model on resting-state and emotionally charged EEG-based FC features, achieving classification accuracies of 92.3%, 94.9%, and 89.7%. Metin et al. (2024) combined 1D-CNN with LSTM and 2D-CNN to classify bipolar disorder, reporting a higher accuracy (95.91%) with the 2D-CNN compared to the 1D-CNN+LSTM (93%). de et al. (2024) proposed SLiTRANet, a transformer-based DL framework for MDD detection, achieving 99.92% accuracy, 99.90% sensitivity, and 99.95% specificity. Zhu et al. (2025) introduced MTNet, a transformer network integrating EEG and eye-tracking data for depression detection, obtaining 91.79% accuracy and highlighting the benefits of intermediate fusion. Ahmed et al. (2024) utilized an ensemble of transformer based models (vanilla BERT, BERTweet, ALBERT) to classify depression severity from social media posts, while Ilias et al. (2024) employed BERT and MentalBERT with extra-linguistic information for depression and stress detection. Sun et al. (2023) introduced TensorFormer, a multimodal transformer framework for sentiment analysis and depression detection, demonstrating performance enhancements on multiple datasets.

Decentralized learning approaches such as FL have also garnered attention. Zhang et al. (2023) proposed FedBrain for diagnosing brain disorders, integrating data augmentation, domain alignment, and personalized predictors to handle high-dimensional features and variable data distributions. FedBrain achieved 79% accuracy with privacy preservation through differential privacy and homomorphic encryption. Li et al. (2023) introduced CAFed, an asynchronous federated CNN-based optimizer for detecting depression from social media data, improving communication efficiency, convergence rates, and privacy protection while surpassing FedAvg in non-convex problem settings.

Although these studies demonstrate promising performance, their reliance on traditional ML and DL methods often involves centralized or FL-based architectures that either risk privacy or suffer from resource constraints. Therefore, this work adopts SL as a resource-sharing methodology to address these concerns, balancing privacy preservation with computational feasibility.

## 3 Materials and methods

**Figure 1:**
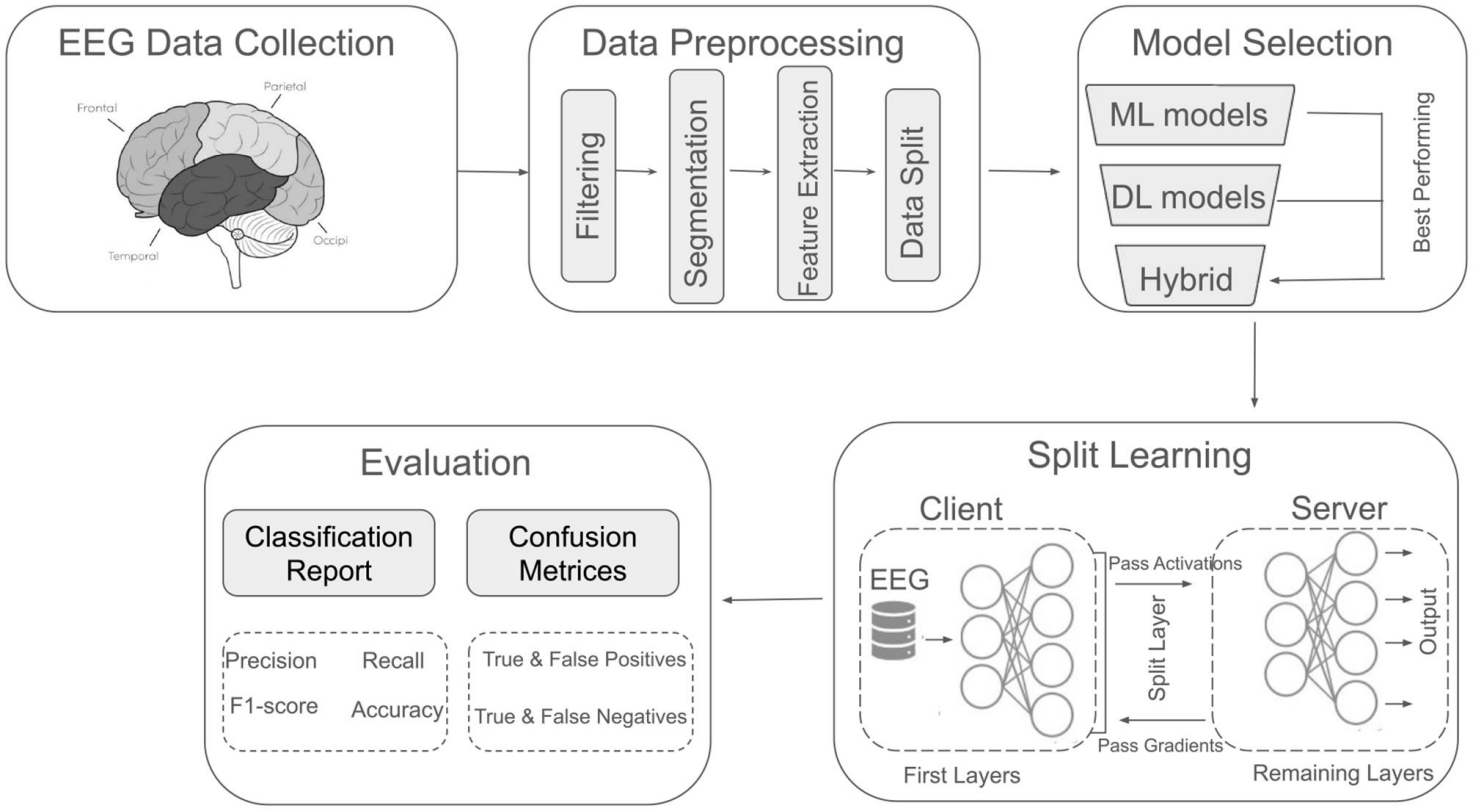
Overview of the utilized methodology for major depressive disorder classification using EEG signals.

This section describes the experimental procedures and methods employed in this study. Figure 1 presents an overview of the methodology, which comprises five key components: EEG data collection, data preprocessing, model selection, SL, and evaluation. Each component is discussed in detail in the subsequent subsections.

### 3.1 Data collection

**Figure 2:**
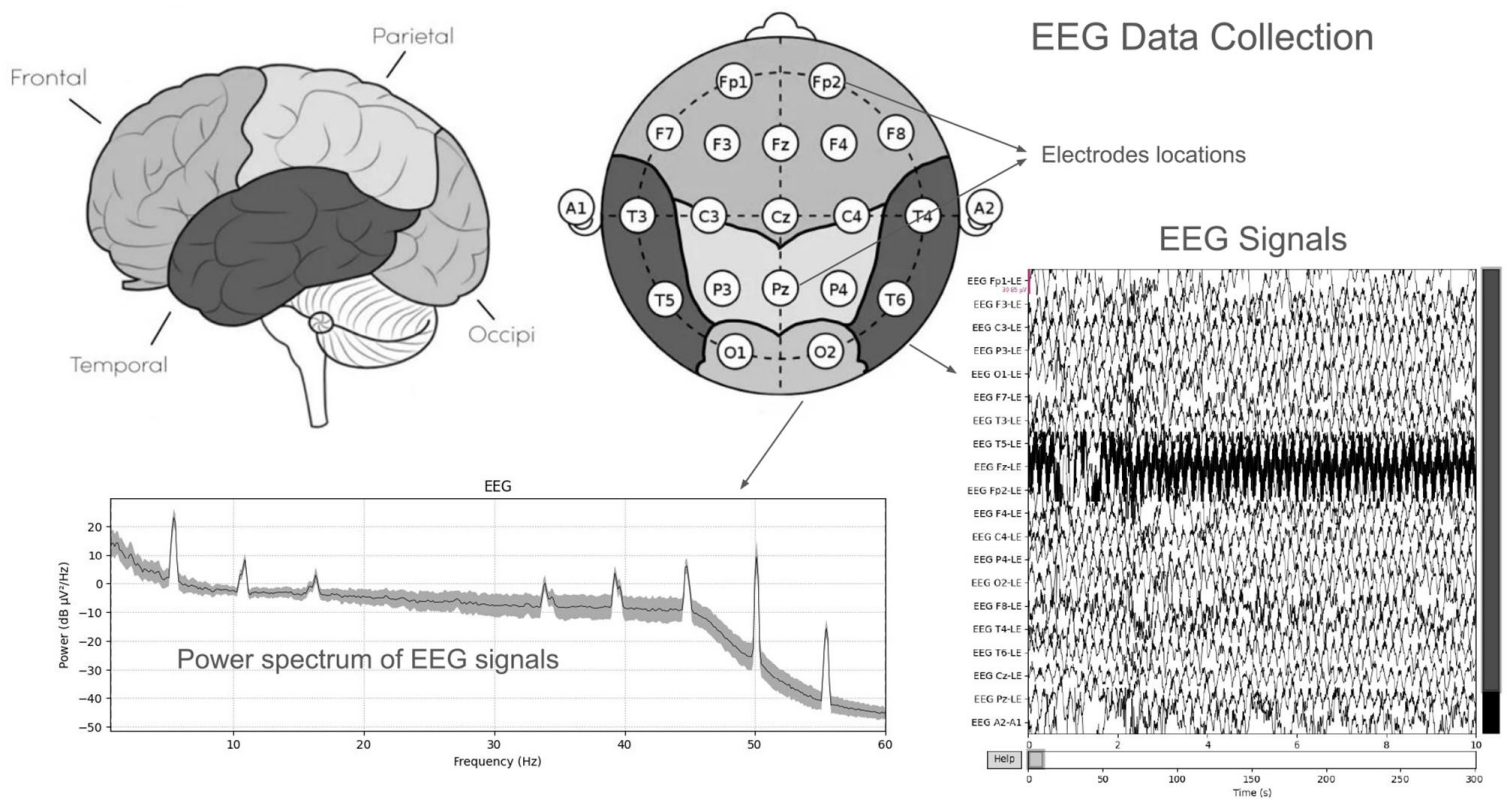
EEG Data collection using electrodes across various locations.

**Figure 3:**
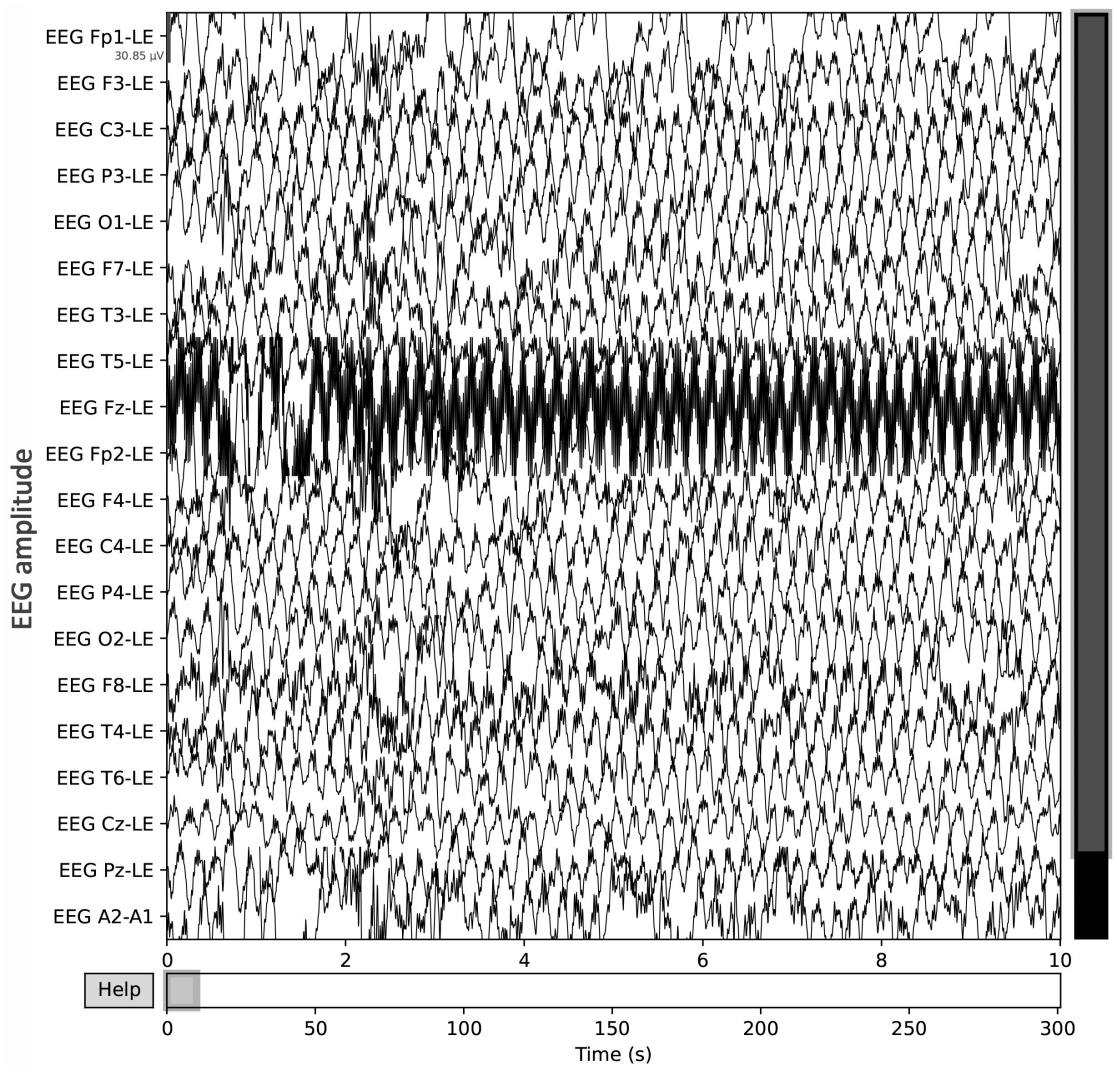
Raw EEG data, recorded over 19 channels in a 10-second window, demonstrates the time-domain structure of brain activity.

**Figure 4:**
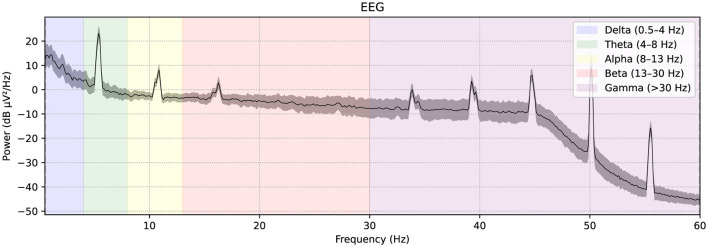
The power spectral density of the EEG signal, color-coded to highlight the standard frequency bands: Delta (0.5–4 Hz), Theta (4–8 Hz), Alpha (8–13 Hz), Beta (13–30 Hz), and Gamma (>30 Hz).

A publicly available EEG dataset (Mumtaz, 2016) is used in this study, comprising of two groups: 33 MDD patients (mean age 40.33 ± 12.86) and 30 age-matched healthy controls (mean age 38.23 ± 15.64), recruited from the outpatient clinic at Hospital Universiti Sains Malaysia (HUSM) (Mumtaz et al., 2017). EEG data were recorded under controlled conditions, with 5-min eyes-closed (EC) and eyes-open (EO) sessions, using a 19-channel system aligned with the international 10–20 standard and a linked-ear reference (Figure 2). The system applied a 0.5–70 Hz bandpass filter, a 50 Hz notch filter, and a sampling rate of 256 Hz, followed by referencing to an infinity reference for subsequent analyses (Mumtaz et al., 2017). Participants were instructed to avoid caffeine and other substances because caffeine intake can alter arousal states by inhibiting adenosine, thus introducing variability and potential noise into EEG recordings (Lesar et al., 2025; Zhu et al., 2024). MDD severity was assessed using the Beck Depression Inventory-II (BDI-II) and the Hospital Anxiety and Depression Scale (HADS) (Mumtaz et al., 2017). A sample shown in Figure 3 of a raw EEG signal recorded over 19 channels in a 10-second window, demonstrates the time-domain structure of brain activity. Accessed dataset (Mumtaz et al., 2017) contains the files structure in pdf format, thus, we utilized python library [i.e., mne (Gramfort et al., 2013)] in order to preprocess these EEG recording for our case, Figure 3 is basically the recordings of EEG sample that is preprocessed via MNE library. Each channel corresponds to a specific scalp location following the international 10–20 system (e.g., Fp1, F3, P3), allowing for regional analysis of cortical oscillations. Notable fluctuations in amplitude can be seen across channels, which may reflect ongoing cognitive or physiological processes, as well as potential artifacts (e.g., eye blinks or muscle movements). Similarly, in Figure 4, the power spectral density of the EEG signal, color-coded to highlight the standard frequency bands: Delta (0.5–4 Hz), Theta (4–8 Hz), Alpha (8–13 Hz), Beta (13–30 Hz), and Gamma (>30 Hz). The PSD curve represents the distribution of signal power across frequencies, with characteristic peaks often observed in the Delta and Alpha ranges. Identifying the relative power in these frequency bands can reveal important information about the participant's mental state and the presence of any abnormal patterns indicative of neurological or psychiatric conditions.

### 3.2 Data preprocessing

#### 3.2.1 Data loading

All EEG recordings were loaded in a standardized manner to ensure uniform data handling. A common input of 10 seconds of EEG recording was used from each sample was then applied across all channels to facilitate consistent inter-subject comparisons.

#### 3.2.2 Filtering

Filtering is an essential step in EEG signal processing because raw signals often contain noise and artifacts in frequency ranges that are not relevant for subsequent analysis. To address this, we employed a bandpass filter to removes unwanted noise and keeping frequency components as well. A bandpass filter in the 0.5–60 Hz range was employed to suppress low-frequency drifts and high-frequency noise. Mathematically, it shown in Equation 1, where *x*(*t*) denotes the raw EEG signal and filtered signal is denoted as $\tilde{x}\left(t\right)$


(1) $$\begin{array}{l} \tilde{x}\left(t\right)=F^{-1}\left\{F\left\{x\left(t\right)\right\}\cdot H\left(\omega \right)\right\} \end{array}$$

Here, F denotes the Fourier transform, and *H*(ω) is the ideal passband response for the specified frequency range.

#### 3.2.3 Epoch segmentation

We segmented the continuous EEG into fixed-length epochs of 5 seconds each, with a 1 second overlap between consecutive segments. This specific window length strikes a practical balance between capturing relevant EEG frequency components (e.g., alpha, beta, and gamma bands) and maintaining adequate temporal resolution for classification. Shorter windows (2–3 seconds) often fail to capture stable patterns, while substantially longer windows (e.g., 8–10 seconds) risk smoothing out important transient features. The 1 second overlap ensures continuity across segment boundaries and mitigates the loss of transitional information that can occur at strict epoch boundaries. Mathematically it is given in Equation 2.


(2) $$E_{i}=\{x(t)|t\in [i\cdot \Delta ,\;(i\cdot \Delta +\tau )]\}$$

where τ = 5 seconds is the epoch length, and Δ = τ−1 seconds denotes the shift applied between consecutive segments.

#### 3.2.4 Feature extraction

Each epoch was transformed into a feature vector by computing a set of statistical descriptors that capture both amplitude variations and higher-order properties of the signal distribution. If *x*_*n*_ denotes the amplitude of the signal at time index *n*, and *N* is the number of samples per epoch, the following examples (using Equations 3–eq6) illustrate key feature computations. Whereas, P2P in Equation 5 refers the peak to peak amplitude of the recorded EEG signal.


(3) $$\begin{array}{l} \mu =\frac{1}{N}\overset{N}{\underset{n=1}{\sum }}x_{n} \end{array}$$

(4) $$\begin{array}{l} \sigma =\sqrt{\frac{1}{N}\overset{N}{\underset{n=1}{\sum }}{\left(x_{n}-\mu \right)}^{2}} \end{array}$$

(5) $$\begin{array}{l} \text{P}2\text{P}=\max\left\{x_{n}\right\}-\min\left\{x_{n}\right\} \end{array}$$

(6) $$\begin{array}{l} \text{RMS}=\sqrt{\frac{1}{N}\overset{N}{\underset{n=1}{\sum }}x_{n}^{2}} \end{array}$$

Higher-order moments, including skewness and kurtosis, were also evaluated to account for asymmetry in the signal distribution.

#### 3.2.5 Labeling

Each epoch was then assigned a class label based on the participant's diagnostic status (0 for healthy controls, 1 for MDD). The final output of this preprocessing pipeline was a feature matrix of size along with a corresponding label vector. This structured dataset was then used for the model training and evaluation.

### 3.3 ML and DL models

**Figure 5:**
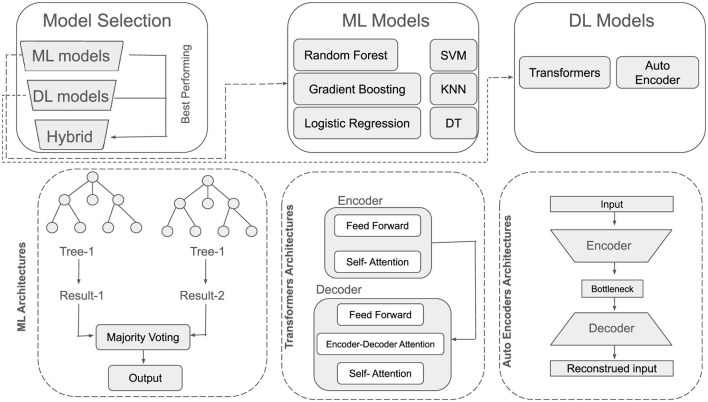
ML and DL models architectural overview.

In this section, the architectures of utilized ML and DL models has been discussed. An overview of their architecture has been shown in Figure 5.

#### 3.3.1 Machine learning classifiers

Model selection plays a pivotal role in achieving robust classification performance. Consequently, the following tree-based and other conventional ML classifiers were employed: LR, RF, SVM, DT, KNN, and GB. Each classifier offers distinct inductive biases and learning strategies that are used to capture diverse patterns in EEG-based features for distinguishing MDD patients from healthy controls.

Moreover, all hyperparameter settings (e.g., n_estimators = 100 for RF, max_depth = 10 for DT, n_neighbors = 7 for KNN) were determined via a grid search procedure. This involved systematically varying key parameters within predefined ranges and evaluating model performance through cross-validation on the training set. The final configurations were selected based on their classification report.

##### 3.3.1.1 Decision tree

A DT recursively partitions the feature space by selecting optimal split points that maximize homogeneity in the resulting subsets. As given in Equation 7, *D* represent the training dataset and *j* be the index of a potential split on feature *x*_*j*_. The split criterion can be based on information gain or the Gini index. For instance, using the Gini index *G*, the split *s* on feature *x*_*j*_ is chosen to minimize.


(7) $$\begin{array}{l} s^{*}=\underset{s}{\text{arg min}}\left[\frac{n_{L}}{n}G\left(D_{L}\right)\;+\;\frac{n_{R}}{n}G\left(D_{R}\right)\right], \end{array}$$

where *D*_*L*_ and *D*_*R*_ are the left and right child partitions of *D* after the split *s*, *n*_*L*_ and *n*_*R*_ are the respective sizes of these partitions, and *n* is the total number of samples in *D*.

##### 3.3.1.2 Random forest

RF constructs an ensemble of decision trees, each trained on a bootstrap sample of the original dataset. At each split node, a random subset of features is considered to enhance diversity among the trees. The model's prediction is obtained via majority voting (for classification) across all trees. Mathematically it is given in Equation 8.


(8) $$\hat{y}=\text{mode}(\{h_{t}(x)|t=1,\ldots ,\;T\}),$$

where *h*_*t*_(**x**) denotes the prediction from the *t*-th tree and *T* is the total number of trees in the forest.

##### 3.3.1.3 Gradient boosting

GB sequentially fits new weak learners (often decision trees) to the negative gradient of a specified loss function. As given in Equation 9, *y*_*i*_ denotes the true label of instance *i*, and let *F*_*m*−1_ be the ensemble model at iteration (*m*−1). A new base learner *h*_*m*_ is trained to approximate the negative gradient of the loss ℓ(*y*_*i*_, *F*_*m*−1_(**x**_*i*_)). The ensemble is then updated as:


(9) $$\begin{array}{l} F_{m}\left(\text{x}\right)=F_{m-1}\left(\text{x}\right)+\eta \cdot h_{m}\left(\text{x}\right), \end{array}$$

where η is the learning rate. This iterative procedure allows optimizer to correct the residual errors from the previous step, leading to improved performance over single-tree methods.

##### 3.3.1.4 Logistic Regression

LR estimates the probability that a sample **x** belongs to the positive class (denoted by *y* = 1) using the sigmoid function. As given in Equation 10:


(10) $$\begin{array}{l} p\left(\text{x}\right)=\sigma \left(\beta^{⊤}\text{x}\;+\;\beta_{0}\right)=\frac{1}{1+\exp\left(-\left(\beta^{⊤}\text{x}\;+\;\beta_{0}\right)\right)}, \end{array}$$

where *β* is the weight vector, β_0_ is the intercept, and σ(·) represents the sigmoid. A threshold (i.e., 0.5) is applied to *p*(**x**) to determine class of the given input.

##### 3.3.1.5 Support vector machine

SVM is a widely used supervised learning technique renowned for its effectiveness in high-dimensional spaces and robust generalization capabilities. The key principle of SVM lies in finding an optimal decision boundary (hyperplane) that maximizes the margin between different classes, thus improving classification performance. In its linear form, SVM is given in Equation 11,


(11) $$\begin{array}{l} \underset{\text{w},b}{\text{minimize}}\frac{1}{2}||\text{w}|{|}^{2}\text{ subject to }y_{i}\left({\text{w}}^{⊤}{\text{x}}_{i}+b\right)\geq 1,\forall i, \end{array}$$

where **w** and *b* define the hyperplane, and *y*_*i*_∈{−1, +1} denotes class labels. Nonlinear decision boundaries can be learned via kernel functions.

##### 3.3.1.6 K-Nearest Neighbors

K-Nearest Neighbors (KNN) is a simple yet effective non-parametric, instance-based learning method. It assigns a class to a query point **x**_*q*_ by considering the classes of its *k* nearest neighbors. The distance metric often used is the Euclidean distance (as given in Equation 12).


(12) $$\begin{array}{l} d\left({\text{x}}_{q},{\text{x}}_{i}\right)=\sqrt{\overset{M}{\underset{j=1}{\sum }}{\left(x_{qj}-x_{ij}\right)}^{2}}, \end{array}$$

where *M* is the number of features. The predicted class is determined by a majority vote among these *k* neighbors.

### 3.4 DL models

#### 3.4.1 Transformer models

Transformer architectures have gained prominence for their capacity to capture long-range dependencies and context within sequential data, making them particularly appealing for EEG-based analysis. Unlike traditional recurrent networks, Transformers dispense with explicit recurrence and convolutional operations, relying instead on an attention mechanism. Mathematically (as shown in Equation 13), *Q*, *K*, and *V* denote the query, key, and value matrices, respectively, then a single-head attention module can be written as:


(13) $$\begin{array}{l} \text{Attention}\left(Q,K,V\right)=\text{softmax }\left(\frac{QK^{⊤}}{\sqrt{d_{k}}}\right)V, \end{array}$$

where *d*_*k*_ is the dimension of the key vectors, and softmax function normalizes the attention scores. Multi-head attention extends this formulation by employing several parallel attention mechanisms and concatenating their outputs to enrich the representational capacity (as given in Equation 14).


(14) $$\text{MultiHead}(Q,K,V)\;=\;{\|}_{h=1}^{H}\text{Attention}(QW_{h}^{Q},\;KW_{h}^{K},\;VW_{h}^{V})W^{O},$$

where || denotes concatenation across *H* attention heads, and $W_{h}^{Q}$, $W_{h}^{K}$, $W_{h}^{V}$, and *W*^*O*^ are learned projection matrices.

In EEG analysis, input sequences can be framed as embeddings of multi-channel signals over time, enabling the transformer to learn context-dependent patterns relevant for mental health classification. Positional encodings are commonly added to the input embeddings to preserve temporal order. This attention-based approach often yields superior performance in capturing nuanced dependencies within EEG signals, especially for tasks such as MDD detection.

#### 3.4.2 Autoencoders

Autoencoders are a family of neural network models designed to learn compressed representations (encodings) of the input data by minimizing reconstruction error. They consist of two main components i.e., Encoder and Decoder. Encoder, maps an input **x**∈ℝ^*D*^ to a latent code **z**∈ℝ^*d*^ (with *d*<*D*) as given in Equation 15,


(15) $$\begin{array}{l} \text{z}=f_{\text{enc}}\left(\text{x}\right). \end{array}$$

Whereas, decoder reconstructs the original input from **z**, producing $\hat{\text{x}}\in {\mathbb{R}}^{D}$ as when in Equation 16:


(16) $$\begin{array}{l} \hat{\text{x}}=f_{\text{dec}}\left(\text{z}\right). \end{array}$$

The model is typically optimized to minimize:


(17) $$\begin{array}{l} L=||\text{x}-\hat{\text{x}}|{|}^{2}, \end{array}$$

another suitable measure of reconstruction fidelity. By constraining the latent dimension *d*, autoencoders learn salient features that represent the most informative aspects of the data. In EEG-based MDD detection, autoencoders can help denoise signals or extract meaningful representations that capture underlying neural patterns. These learned representations may then serve as inputs for downstream classifiers or be integrated into end-to-end DL pipelines for improved diagnostic accuracy.

### 3.5 Ensemble learning

Ensemble learning combines multiple base models to achieve improved predictive performance relative to any single constituent model. This approach capitalizes on the principle of “wisdom of the crowd,” where diverse model outputs are aggregated to form a final decision. A common strategy for building ensembles include:

#### 3.5.1 Bagging

Bagging (Bootstrap Aggregating) trains each base learner on a different bootstrap sample (randomly drawn with replacement) of the original dataset. Let ${\left\{D_{b}\right\}}_{b=1}^{B}$ be the collection of bootstrap samples, each used to train a distinct model *h*_*b*_(**x**). The final prediction is obtained by averaging or voting across the ensemble:


(18) $${\hat{y}}_{\text{bagging}}\;=\;\{\begin{array}{ll} \text{majority}{\left\{h_{b}(x)\right\}}_{b=1}^{B}, & \text{classification}\\ \frac{1}{B}\sum_{b=1}^{B}h_{b}(x), & \text{regression} \end{array}$$

Bagging often reduces variance without substantially increasing bias, making it effective for high-variance models like decision trees.

Ensemble learning is particularly relevant for EEG-based MDD classification due to the high dimensionality and variability inherent in EEG signals. It is because of this reason, ensemble learning was utilized using best performing ML model and then best performing DL model. By integrating these models, ensembles have the potential to yield more reliable and generalizable predictions for clinical applications.

### 3.6 Split learning

**Figure 6:**
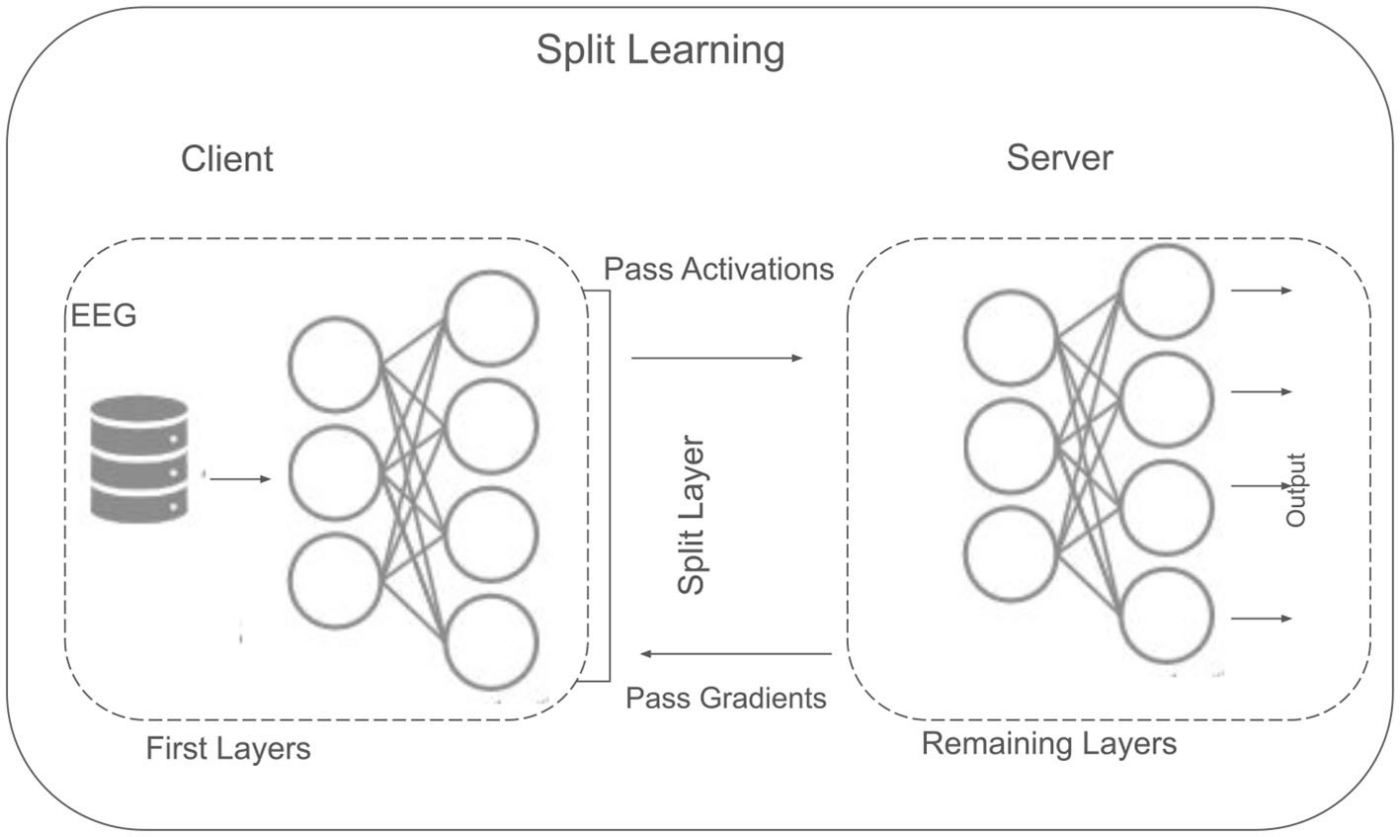
Split learning concept.

SL offers a decentralized framework designed to address privacy and resource constraints, particularly relevant when clinical or EEG datasets cannot be shared in raw form. Unlike fully centralized methods, where all data must reside on a single server, SL divides a neural network into multiple segments to be trained collaboratively between clients and a central server. In this study, three clients are assumed, each holding a portion of the EEG data locally (as shown in Figure 6). After data preprocessing (Section 3.2), SL is implemented to enable model training without direct data exchange across clients.

#### 3.6.1 Architectural overview

SL offers a collaborative training framework by partitioning a neural network between clients and a central server. This approach helps ensure that sensitive data like EEG signals remain local to each client, while still enabling the development of robust, shared models. In the context of MDD classification, SL architecture that we used is shown in Figure 6 that is particularly beneficial, as it enable data training while managing resources efficiently.Consider a neural network *f*(·) decomposed into two primary segments (as given in Equation 19. Where: *f*_client_ denotes the partial model residing on the client side, parametrized by *θ*, which transforms local data **x** into an intermediate representation ***z***. *f*_server_ denotes the remaining portion of the model, located on a central server and parametrized by *ϕ*. It processes the intermediate representation ***z*** to produce predictions (e.g., class probabilities). And, ◇ symbolizes the functional concatenation of the two segments.


(19) $$\begin{array}{l} f\left(\text{x};\theta ,\phi \right)=f_{\text{client}}\left(\text{x};\theta \right)◇f_{\text{server}}\left(z;\phi \right) \end{array}$$

Each client trains only *f*_client_ on its local dataset, while *f*_server_ is trained on the server side using the intermediate representations ***z*** received from the clients. This design ensures that raw EEG data never leaves the client's local environment. In utilized methodology for SL, each client *i* forwards only intermediate activations ***z*** derived from its local data to the server, which handles the remaining layers and calculates the global loss. The server's gradients are backpropagated to the clients, enabling local updates while preserving data privacy. This division of computational labor also alleviates resource constraints on client devices, as the heaviest computations can be offloaded to the server. This makes SL particularly applicable for MDD classification, where healthcare institutions typically hold proprietary EEG data. By sharing only intermediate features, SL mitigates privacy concerns and fosters collaborative model development, enabling a more inclusive and robust system for detecting and monitoring mental health conditions.

#### 3.6.2 Algorithmic workflow for split learning

In this subsection, workflow of the utilized SL methodology has been described, as it starts with initialization, local processing and then toward clients processing and propagation, these steps are given as below:

##### 3.6.2.1 Initialization

Each client *C*_*i*_ initializes its local model parameters *θ*_*i*_, while the central server initializes its parameters *ϕ*. Data normalization or other preliminary setup is performed here.

##### 3.6.2.2 Local preprocessing

Prior to training, each client cleans and preprocesses its local EEG data (e.g., filtering, artifact removal). This ensures high-quality input to the client-side model *f*_client_(·;*θ*_*i*_).

##### 3.6.2.3 Client forward pass

The client-side model *f*_client_ processes the local EEG data *D*_*i*_ to produce intermediate representations ***z***_*i*_. Because only ***z***_*i*_ is shared, raw EEG data remains private.

##### 3.6.2.4 Intermediate transmission

Clients transmit ***z***_*i*_ to the central server. This step preserves data privacy, as the raw EEG signals never leave the local environment.

##### 3.6.2.5 Server forward pass and loss computation

The central server processes all received activations {***z***_*i*_} using The server computes a global loss *L* by aggregating individual losses (e.g., cross-entropy) for each client's predictions *y*_*i*_.

##### 3.6.2.6 Backpropagation and parameter updates

Using the global loss *L*, the server performs backpropagation to update its parameters *ϕ*. By the chain rule, partial gradients are also computed and sent back to each client.

##### 3.6.2.7 Client-side parameter updates

Upon receiving the relevant gradients, each client updates its local parameters *θ*_*i*_. This allows clients to learn collaboratively without ever sharing raw EEG data.

##### 3.6.2.8 Iteration and convergence

All previous steps (from local preprocessing to parameter updates) are repeated for multiple epochs. Once convergence is reached, the final model consists of updated client-side parameters {*θ*_*i*_} and server-side parameters *ϕ*.

##### 3.6.2.9 Output

The trained SL model can be deployed for EEG classification. Each client retains its local model segment *θ*_*i*_, while the server holds *ϕ*, ensuring continual privacy protection.

### 3.7 Evaluation metrics

Classification performance was evaluated using standard metrics derived from the confusion matrix in a binary classification setting (Healthy vs. MDD). Let TP (True Positive) be the number of MDD instances correctly classified, TN (True Negative) the number of Healthy instances correctly classified, FP (False Positive) the number of Healthy instances misclassified as MDD, and FN (False Negative) the number of MDD instances misclassified as Healthy. These values form the following 2 × 2 confusion matrix, from this matrix, the evaluation metrics are computed that are accuracy, precision, recall, and F1-score:


$$\left[\begin{array}{cc} \text{TP} & \text{FP}\\ \text{FN} & \text{TN} \end{array}\right]$$

#### 3.7.1 Accuracy

Accuracy (Equation 20) measures the overall rate of correct predictions across all instances. It is the proportion of TP and TN from all predicted values by the model. It measures the proportion of instances that are correctly predicted out of the total number of predictions.


(20) $$\begin{array}{l} \text{Accuracy}=\frac{\text{TP }+\text{ TN}}{\text{TP }+\text{ TN }+\text{ FP }+\text{ FN}}. \end{array}$$

#### 3.7.2 Precision

Precision is a crucial metric that quantifies the model's ability to correctly identify positive (MDD) cases among all predicted positives. As given in Equation 21, it is the ratio of TP to the sum of FP and TN


(21) $$\begin{array}{l} \text{Precision}=\frac{\text{TP}}{\text{TP }+\text{ FP}}. \end{array}$$

#### 3.7.3 Recall

Recall, sometimes referred to as sensitivity, measures the model's effectiveness at identifying all positive (MDD) instances in a dataset. Mathematically, as given in Equation 22, it is the ratio of TP to the sum of TN and FN.


(22) $$\begin{array}{l} \text{Recall}=\frac{\text{TP}}{\text{TP }+\text{ FN}}. \end{array}$$

#### 3.7.4 F1-Score

The F1-Score provides a balanced assessment of a model's performance by combining both Precision and Recall into a single metric. Mathematically expressed in Equation 23, it is the harmonic mean of Precision and Recall. Unlike a simple arithmetic mean, the harmonic mean penalizes extreme values, ensuring both Precision and Recall share comparable significance in the final score.


(23) $$\begin{array}{l} \text{F}1\text{ Score}=2\times \frac{\text{Precision }\times \text{ Recall}}{\text{Precision }+\text{ Recall}}. \end{array}$$

#### 3.7.5 Confusion matrix

Confusion matrix provides a visual overview of classification performance. It indicates how frequently the classifier confuses one class for the other, offering deeper insight into errors (FPs vs. FNs). For binary classification (Healthy vs. MDD), the matrix aids in diagnosing misclassification patterns and refining model strategies. All these metrics collectively form the classification report, enabling a comprehensive assessment of each model's performance in detecting MDD from EEG signals.

## 4 Results and discussion

In this section, we present a comprehensive evaluation of the proposed classification approaches for MDD detection. We analyze the performance of both ML and DL models, and additionally showcase an ensemble method that utilizes the SL framework. By assessing metrics such as accuracy, precision, recall, and F1-Score, we gain insight into each model's strengths and limitations.

### 4.1 ML models results

As discussed earlier, several ML models i.e., LR, RF, SVM, DT, KNN, and GB were utilized to classify MDD using EEG data. Table 1 presents their respective performances on the test set, along with best cross-validation (CV) scores and optimal hyperparameter configurations. The key findings for each model are summarized below.

**Table 1:** Performance of Various Machine Learning Models for MDD Classification.

**Model**	**Best score (CV)**	**Best params**	**Accuracy**	**F1 (Healthy)**	**F1 (MDD)**	**TP**	**FP**	**FN**	**TN**
Logistic regression	0.8833	{C: 0.1}	0.9241	0.9160	0.9308	9,382	1,022	374	7,609
Random forest	0.9138	{max_depth: None, n_estimators: 100}	1.0000	1.0000	1.0000	9,756	0	0	8,631
SVM	0.9182	{C: 10, kernel: rbf}	0.9874	0.9865	0.9882	9,699	175	57	8,456
Decision tree	0.8740	{max_depth: 10}	0.9775	0.9759	0.9790	9,606	263	150	8,368
K-Nearest Neighbors	0.8713	{n_neighbors: 7, weights: distance}	1.0000	1.0000	1.0000	9,756	0	0	8,631
Gradient Boosting	0.9184	{learning_rate: 0.2, n_estimators: 100}	0.9935	0.9931	0.9939	9,721	84	35	8,547

#### 4.1.1 LR model

Achieved a test accuracy of 92.41%, with F1-Scores of 0.9160 for the Healthy class and 0.9308 for the MDD class. Its best CV score was 0.8833. These results suggest that LR provides a stable generalization capability when distinguishing between Healthy and MDD samples. The best hyperparameter setting at C: 0.1 indicates a preference for regularization to control overfitting in high-dimensional EEG feature spaces.

#### 4.1.2 RF model

**Figure 7:**
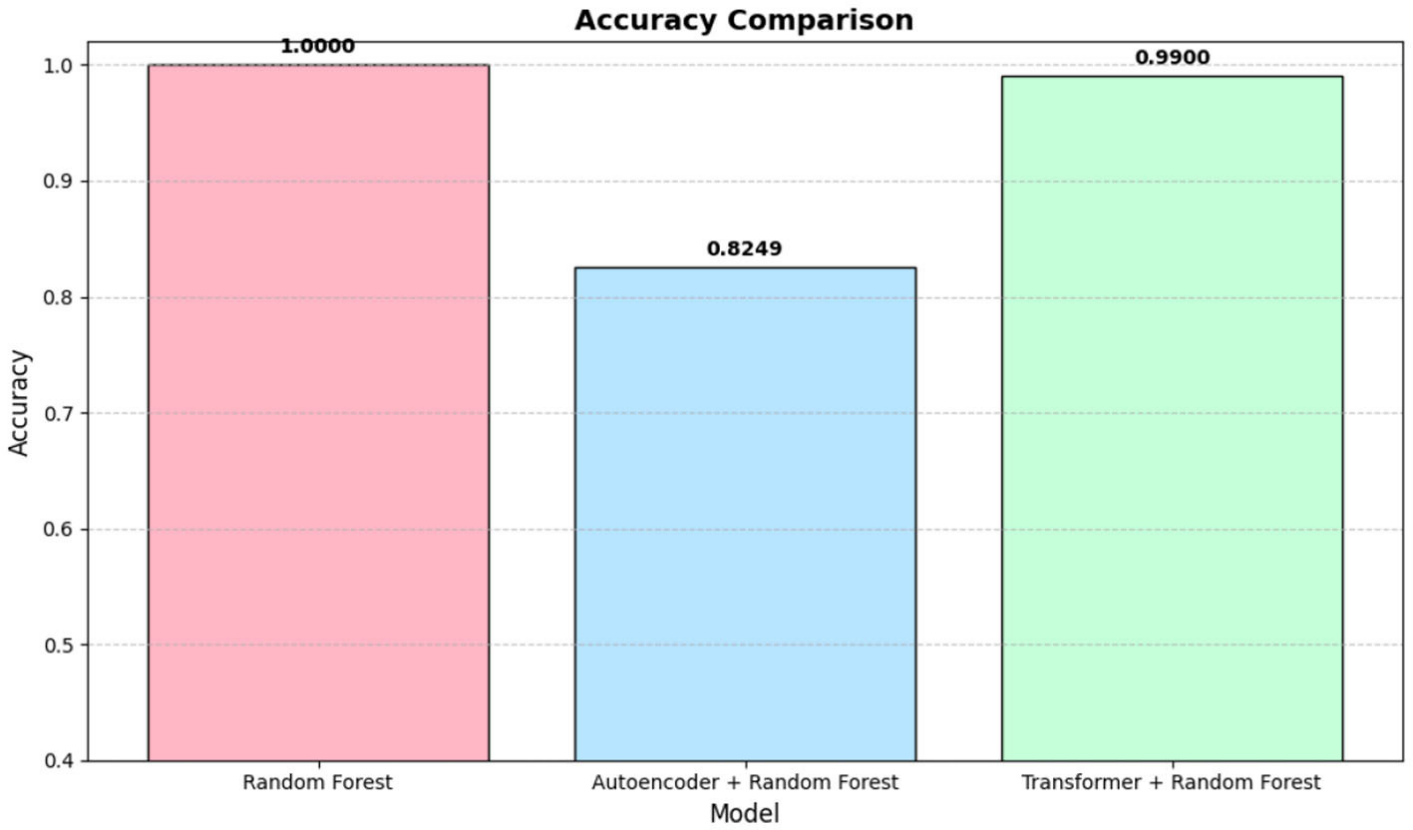
Accuracies comparison for best performing ML along with ensemble DL model.

Achieved a test accuracy of 100%, outperforming other ML models. Its best CV score was 0.9138. The selected hyperparameter (number of estimators 100) enable an ensemble of sufficiently large and diverse trees. Due to its strong performance, RF was chosen for the ensemble approach with Deep Learning models, as shown in Figure 7.

#### 4.1.3 SVM model

Achieved an accuracy of 98.74%, indicating a clear separation between the two classes. Its F1-Scores of 0.9865 (Healthy) and 0.9882 (MDD) reflect the model's effectiveness. The best CV score was 0.9182, achieved with hyperparameter (C: 10, kernel: rbf). This shows that SVM is suitable for handling EEG data with potentially complex class boundaries.

#### 4.1.4 DT model

Achieved an accuracy of 97.75%. Its best CV score was 0.8740. By employing a moderately deep tree with max depth of 10, the DT model partitions the EEG feature space effectively. Although decision trees can overfit, this depth appears to balance training accuracy and generalization for the MDD classification task.

#### 4.1.5 KNN model

Achieved an accuracy of 100%, similar to the RF model. Its best CV score was 0.8713. The chosen hyperparameters number of neighbors: 7, weights: distance uses distance-based weighting in separable EEG clusters. However, KNN can be computationally expensive at inference time and typically requires extensive parameter tuning for integration with DL pipelines, so it was not selected for the ensemble stage.

#### 4.1.6 GB model

Achieved an accuracy of 99.35%, with a best CV score of 0.9184. It iteratively refined weak learners using a learning rate of 0.2 and 100 estimators. Its F1-Scores of 0.9931 (Healthy) and 0.9939 (MDD) indicate that boosting rounds improve classification by reducing both bias and variance.

Table 1 shows that all models attain high classification performance. RF and KNN reach 100% accuracy on the test set, while SVM, DT, LR, and GB also present strong results. The consistent F1-Scores reinforce the effectiveness of EEG features for detecting MDD.

#### 4.1.7 K Fold cross validation results

Table 2 shows the accuracy for each classifier across four folds of cross-validation. The Mean column reports the average accuracy across all folds. By separating the data into distinct training/validation splits for each fold, we reduce the risk of overfitting and obtain a more realistic estimate of out-of-sample performance.

**Table 2:** 4-Fold cross-validation accuracies for each classifier.

**Model**	**Fold 1**	**Fold 2**	**Fold 3**	**Fold 4**	**Mean**
Logistic regression	0.92	0.88	0.93	0.90	0.91
Random forest	1.00	0.99	0.96	1.00	0.98
SVM	0.95	0.94	0.98	0.97	0.96
Decision tree	0.87	0.88	0.90	0.86	0.88
K-Nearest Neighbors	0.99	0.96	0.95	0.98	0.97
Gradient boosting	0.93	0.94	0.95	0.92	0.93

### 4.2 DL models performances along with ensemble learning

After training an autoencoder to learn compact EEG representations, multiple classifiers were evaluated on these latent features. Table 3 summarizes the results for both a baseline autoencoder-only ensemble and five conventional ML algorithms trained on autoencoder outputs. Each row reports the overall accuracy as well as precision, recall, and F1-scores for both classes (Healthy and MDD).

**Table 3:** Classification performance on autoencoder and with ensemble autoencoder.

**Method**	**Accuracy**	**Healthy**			**MDD**		
		**Precision**	**Recall**	**F1**	**Precision**	**Recall**	**F1**
**Autoencoder (baseline)**	0.6884	0.7031	0.5817	0.6367	0.6791	0.7828	0.7273
**Autoencoder** **+** **random forest**	0.8249	0.9321	0.6761	0.7837	0.7696	0.9565	0.8529
**SVM**	0.8222	0.9061	0.6929	0.7853	0.7752	0.9365	0.8483
**Autoencoder** **+** **decision tree**	0.6833	0.6947	0.5800	0.6321	0.6759	0.7746	0.7219
**Autoencoder** **+** **K-Nearest Neighbors**	0.7692	0.7627	0.7375	0.7499	0.7745	0.7971	0.7857
**Autoencoder** **+** **gradient boosting**	0.7735	0.8187	0.6645	0.7336	0.7457	0.8699	0.8030

#### 4.2.1 Discussion of autoencoder-based results

Table 3 demonstrates that using autoencoder-derived representations yield competitive performance across multiple classifiers. The baseline ensemble (first row) provides a moderate accuracy of 0.6884, indicating that unsupervised feature extraction alone captures some discriminative patterns.

RF and SVM show the highest accuracies (over 0.82), suggesting that tree-ensemble and margin-based methods effectively exploit these latent features. K-Nearest Neighbors and Gradient Boosting also achieve an accuracies of approximately 0.77, while the single DT model exhibits lower performance (0.68) relative to ensemble approaches. RF high precision for Healthy (0.9321) and recall for MDD (0.9565) underline its balanced detection capabilities in this context.

#### 4.2.2 Transformer-based classification

As transformer model is utilized to capture long-range dependencies in EEG signals, several classifiers were applied to the Transformer outputs for final predictions as well. Table 4 summarizes the results, including a standalone Transformer baseline and five conventional ML classifiers. The table reports overall accuracy, alongside precision, recall, and F1-scores for the two classes (Healthy vs. MDD). Their detailed results discussion has been given in Section 4.2.3.

**Table 4:** Classification performance on transformer and ensemble models.

**Method**	**Accuracy**	**Healthy**			**MDD**		
		**Precision**	**Recall**	**F1**	**Precision**	**Recall**	**F1**
**Transformer (baseline)**	0.9000	0.9100	0.8800	0.8950	0.9000	0.9200	0.9100
**Transformer** **+** **decision tree**	0.8800	0.8900	0.8600	0.8750	0.8700	0.8900	0.8800
**Transformer** **+** **K-Nearest Neighbors**	0.9200	0.9100	0.9200	0.9150	0.9300	0.9200	0.9250
**Transformer** **+** **SVM**	0.9300	0.9400	0.9200	0.9300	0.9300	0.9400	0.9350
**Transformer** **+** **gradient boosting**	0.9500	0.9500	0.9400	0.9450	0.9400	0.9500	0.9450
**Transformer** **+** **random forest**	0.9900	0.9900	0.9900	0.9900	0.9900	0.9900	0.9900

#### 4.2.3 Discussion of transformer-based results

In this subsection the results achieved for ensemble learning has been discussed, as we utilized transformers along with ML models and these has been given in Table 4 that shows the classification performance of the baseline Transformer model and its combinations with different ML classifiers. The standalone Transformer (Baseline) achieves an accuracy of 0.90, with 0.91 precision, 0.88 recall, and 0.895 F1 for the Healthy class, and 0.90 precision, 0.92 recall, and 0.91 F1 for the MDD class. These results indicate that the Transformer can extract features from EEG signals that help differentiate between Healthy and MDD instances.

**Transformer** **+** **DT** yields an accuracy of 0.88. For the Healthy class, it achieves 0.89 precision, 0.86 recall, and 0.875 F1, while for the MDD class it attains 0.87 precision, 0.89 recall, and 0.88 F1. Even though this is lower than some other combinations, it still shows reasonable performance compared to traditional EEG-based methods.

**Transformer** **+** **KNN** reports an accuracy of 0.92. The Healthy class has 0.91 precision, 0.92 recall, and 0.915 F1, and the MDD class has 0.93 precision, 0.92 recall, and 0.925 F1. These numbers suggest that local distance-based methods can work well when applied to Transformer outputs.

**Transformer** **+** **SVM** achieves an accuracy of 0.93. For the Healthy class, precision, recall, and F1 are 0.94, 0.92, and 0.93, respectively, while for the MDD class they are 0.93, 0.94, and 0.935. This indicates that margin-based classification benefits from sequence-aware features extracted by the Transformer.

**Transformer** **+** **GB** attains an accuracy of 0.95. Its Healthy metrics are 0.95 precision, 0.94 recall, and 0.945 F1, and its MDD metrics are 0.94 precision, 0.95 recall, and 0.945 F1. This suggests that boosting rounds are effective at refining the latent representations provided by the Transformer.

**Transformer** **+** **RF** achieves the highest accuracy of 0.99. Precision, recall, and F1 for both Healthy and MDD classes are all 0.99, showing that the ensemble of decision trees makes good use of attention-based features.

Thus, combining the Transformer with robust classification algorithms enhances performance compared to the baseline. The best results come from pairing the Transformer with RF, followed by GB, SVM, KNN, and DT. These findings illustrate that attention-based feature extraction can improve EEG-based MDD classification when integrated with well-chosen ML methods.

### 4.3 Split learning results

SL framework was implemented across three clients, each training local Transformer-based encoders whose latent representations were subsequently processed by a RF classifier on the server side. Table 5 shows the key performance metrics (Accuracy, Precision, Recall, and F1-Score) for each client, alongside the main confusion matrix values (correct vs. misclassified instances of Healthy and MDD). The average inference time per client was measured at 2.0866 seconds.

**Table 5:** Performance of split learning across three clients.

**Client**	**Accuracy**	**Precision**	**Recall**	**F1-Score**	**Healthy**		**MDD**	
					**Correct**	**Misclass**.	**Correct**	**Misclass**.
**Client 1**	0.9574	0.9577	0.9574	0.9574	2,744	172	3,124	89
**Client 2**	0.9623	0.9625	0.9623	0.9623	2,679	148	3,219	83
**Client 3**	0.9543	0.9549	0.9543	0.9543	2,691	197	3,158	83

To quantify inference time, we define the total inference time for a single sample on the *i*-th client as given in Equation 24:


(24) $$\begin{array}{c} T_{\text{inference}}^{\left(i\right)}=T_{\text{local}}^{\left(i\right)}\;+\;T_{\text{transfer}}^{\left(i\right)}\;+\;T_{\text{server}}, \end{array}$$

where $T_{\text{local}}^{\left(i\right)}$ is the local forward pass time through the Transformer on client *i*, $T_{\text{transfer}}^{\left(i\right)}$ is the latency for transmitting the latent representation to the server, and *T*_server_ is the server-side classification time using the RF model. The average inference time *T*_inference_ across all *k* clients mathematically is given in Equation 25.


(25) $$\begin{array}{c} T_{\text{inference}}=\frac{1}{k}\overset{k}{\underset{i=1}{\sum }}T_{\text{inference}}^{\left(i\right)}. \end{array}$$

Thus got an average *T*_inference_ of 2.0866 seconds. This end-to-end metric reflects the time from when an EEG sample arrives at the client to when the final classification outcome is returned, including both local and server-side computations.

#### 4.3.1 Discussion of split learning results

**Figure 8:**
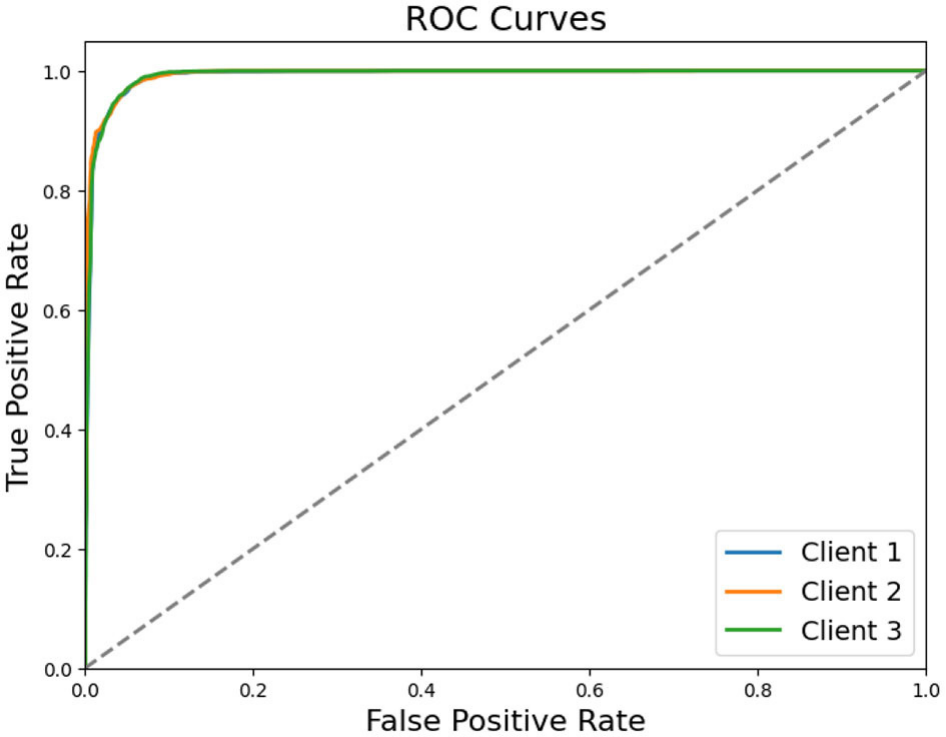
ROC curves for clients in SL settings.

Table 5 illustrates that all three clients attain high classification accuracy, exceeding 95%. Client 2 achieves the best overall accuracy of 0.9623, closely followed by Client 1 (0.9574) and Client 3 (0.9543). Precision and Recall remain closely aligned for each client, reflecting a balanced ability to detect both Healthy and MDD classes. Confusion matrix counts indicate that relatively few Healthy samples are misclassified as MDD and vice versa. ROC curve shown in Figure 8 also reflects that each client achieved higher true positive rate showing their ability and reliability.

These findings shows that a SL approach, utilized with a transformer architecture for local feature extraction and RF model for final classification, can maintain robust performance while preserving data privacy. Additionally, the measured average inference time of 2.0866 seconds per client suggests that this collaborative framework is computationally feasible for real-world EEG based mental health applications.

While these performance metrics are promising, practical deployment on devices with limited compute capabilities (e.g., mobile EEG headsets, embedded healthcare systems) demands additional optimization. Because SL partitions the model into client-side and server-side segments, heavier computations—such as the Transformer's attention blocks—are executed on the server, reducing on-device resource usage. Future work will involve benchmarking these strategies across diverse hardware platforms to quantify improvements in latency, memory use, and power efficiency.

## 5 Conclusion

This work presented an effective methodology for major depressive disorder classification by integrating advanced EEG feature extraction, ensemble models, and split learning to safeguard privacy. In conventional centralized experiments, RF, KNN, and GB achieved commendable performance, while a Transformer-RF ensemble model achieved 99% accuracy. Autoencoder-based feature learning provided notable results, illustrating that unsupervised approaches can be profitably combined with supervised classifiers. Crucially, the split learning implementation validated the feasibility of decentralizing training: three distinct clients each achieved over 95% accuracy, with minimal performance trade-offs relative to centralized schemes. By maintaining data on local nodes and exchanging only intermediate representations, the framework supported institutional privacy requirements while offering robust classification outcomes. Future investigations may include refining model architectures for improved efficiency, exploring additional neurophysiological data modalities, and extending the approach to multi-disorder classification scenarios, thereby broadening the applicability of privacy-preserving, high-performance EEG analytics in clinical settings.

## Data Availability

The original contributions presented in the study are included in the article/supplementary material, further inquiries can be directed to the corresponding author. The datasets analyzed and utilized for this study can be found at DOI: 10.6084/m9.figshare.4244171.v2.
